# In Search of a Dose: The Functional and Molecular Effects of Exercise on Post-stroke Rehabilitation in Rats

**DOI:** 10.3389/fncel.2020.00186

**Published:** 2020-06-25

**Authors:** Fengwu Li, Xiaokun Geng, Christian Huber, Christopher Stone, Yuchuan Ding

**Affiliations:** ^1^China-America Institute of Neuroscience, Luhe Hospital, Capital Medical University, Beijing, China; ^2^Department of Neurology, Beijing Luhe Hospital, Capital Medical University, Beijing, China; ^3^Department of Neurosurgery, Wayne State University School of Medicine, Detroit, MI, United States; ^4^Department of Research and Development Center, John D. Dingell VA Medical Center, Detroit, MI, United States

**Keywords:** ischemia/reperfusion, functional outcome, synaptogenesis, BDNF, TrkB, CREB, HIF-1α

## Abstract

Although physical exercise has been demonstrated to augment recovery of the post-stroke brain, the question of what level of exercise intensity optimizes neurological outcomes of post-stroke rehabilitation remains unsettled. In this study, we aim to clarify the mechanisms underlying the intensity-dependent effect of exercise on neurologic function, and thereby to help direct the clinical application of exercise-based neurorehabilitation. To do this, we used a well-established rat model of ischemic stroke consisting of cerebral ischemia induction through middle cerebral artery occlusion (MCAO). Ischemic rats were subsequently assigned either to a control group entailing post-stroke rest or to one of two exercise groups distinguished by the intensity of their accompanying treadmill regimens. After 24 h of reperfusion, exercise was initiated. Infarct volume, apoptotic cell death, and neurological defects were quantified in all groups at 3 days, and motor and cognitive functions were tracked up to day-28. Additionally, Western blotting was used to assess the influence of our interventions on several proteins related to synaptogenesis and neuroplasticity (growth-associated protein 43, a microtubule-associated protein, postsynaptic density-95, synapsin I, hypoxia-inducible factor-1α, brain-derived neurotrophic factor, nerve growth factor, tyrosine kinase B, and cAMP response element-binding protein). Our results were in equal parts encouraging and surprising. Both mild and intense exercise significantly decreased infarct volume, cell death, and neurological deficits. Motor and cognitive function, as determined using an array of tests such as beam balance, forelimb placing, and the Morris water maze, were also significantly improved by both exercise protocols. Interestingly, while an obvious enhancement of neuroplasticity proteins was shown in both exercise groups, mild exercise rats demonstrated a stronger effect on the expressions of Tau (*p* < 0.01), brain-derived neurotrophic factor (*p* < 0.01), and tyrosine kinase B (*p* < 0.05). These findings contribute to the growing body of literature regarding the positive effects of both mild and intense long-term treadmill exercise on brain injury, functional outcome, and neuroplasticity. Additionally, the results may provide a base for our future study regarding the regulation of HIF-1α on the BDNF/TrkB/CREB pathway in the biochemical processes underlying post-stroke synaptic plasticity.

## Introduction

Exercise therapy has long been considered a promising strategy to ameliorate physical disability after stroke (Saposnik et al., 2016). However, neurological outcomes of post-stroke rehabilitation appear to differ according to the intensity of the exercise regimen that is used (Bell et al., 2015; Xing et al., 2018). Some previous studies have demonstrated that higher intensity exercise may yield better functional recovery and neuroplasticity (Linder et al., 2019; Luo et al., 2019; Andrews et al., 2020), while other studies have suggested that mild exercise results in superior neuroprotection and synaptic plasticity after stroke (Lee et al., 2009; Shih et al., 2013). These conflicting results underscore the principle that exercise intensity is an important determinant of post-stroke neurological outcomes. Therefore, clarifying the mechanisms that underlie an intensity-dependent effect of exercise on neurologic function may help direct the clinical application of exercise-based neurorehabilitation. Currently, these mechanisms have not been fully explored, and are consequently incompletely understood.

Recently, brain-derived neurotrophic factor (BDNF) has become the subject of increasing attention as a possible mediator of the neurological benefits of exercise. BDNF is an abundant growth factor that is involved in activity-induced neuroplasticity and is upregulated in the animal brain by exercise. The regulation of neuroplasticity depends on a complex set of interactions between a variety of neural proteins, including postsynaptic density 95 (PSD-95; Wang et al., 2019), synapsin I (SYN; Pan et al., 2017), growth-associated protein 43 (GAP-43), and microtubule-associated protein (also known as Tau; Biundo et al., 2018; Mercerón-Martínez et al., 2018; Pu et al., 2019). Changes in these neuroplastic factors are related to exercise-induced activation of BDNF (Kim and Leem, 2016; Belviranli and Okudan, 2019). Previous research has suggested a pivotal regulatory role for BDNF and its receptor, BDNF-tyrosine kinase B (TrkB), regarding neuroplasticity after physical exercise (Lee et al., 2018), mediated through the expression of the transcription factor cyclic AMP response element-binding protein (CREB; Hu et al., 2004). Moreover, activation of the BDNF/TrkB/CREB signaling pathway has also been shown to promote functional recovery after stroke (Liu H. et al., 2018). Taken together, these lines of evidence suggest that post-stroke exercise regimens such as the one used in this study may induce neuroplasticity and influence rehabilitative outcomes through the changes they provoke in the BDNF pathway.

Another factor that may be involved in determining the outcomes of post-stroke exercise regimens is hypoxia-inducible factor-1α (HIF-1α). Upregulation of HIF-1α by exercise has been reported to play a role in reducing infarct volumes following ischemia/reperfusion injury (Li C. et al., 2017), and in post-stroke neuroplasticity (Wu et al., 2018). Previous studies demonstrated that HIF-1α also induced the expression of BDNF (Shi et al., 2009; Nakamura et al., 2011; Helan et al., 2014), and thereby promoted neuroplasticity, reduced neuronal death, and improved neurological function in a rat model of ischemic stroke (Chen et al., 2017). HIF-1α has further been shown to stimulate the expression of the TrkB receptor (Martens et al., 2007), and the CREB receptor in various cancer cells (Yu et al., 2020). However, despite this considerable circumstantial evidence, previous studies have not yet explored the effect of HIF-1α on BDNF/TrkB/CREB pathway in improving synaptic plasticity following ischemia/reperfusion injury. Although the present study did not determine this relation, as the first step, we intended to assess the expression of HIF-1α and BDNF/TrkB/CREB proteins following ischemia/reperfusion injury. These results might suggest a potential association of these molecules and provide a base for our future study regarding the regulation of HIF-1α on the BDNF/TrkB/CREB pathway.

## Materials and Methods

### Animals

A total of 150 adult male Sprague–Dawley rats (280–300 g, Vital River Laboratory Animal Technology Company Limited, Beijing, China) were used in this study. The protocol by which they were studied was approved by the Animal Care and Use Committee of Capital Medical University, and the study was conducted following the NIH Guide for the Care and Use of Laboratory Animals. Animals were randomly divided into three groups: middle cerebral artery occlusion (MCAO) without exercise (50), MCAO plus intense treadmill exercise (50), and MCAO plus mild treadmill exercise (50). Both exercise protocols were initiated after 24 h reperfusion, and animals in each group were sacrificed at days 3, 14, and 28 after reperfusion for further biochemical analysis.

### Focal Cerebral Ischemia

The animals were subjected to transient right MCAO according to the method we described previously (Li F. et al., 2019). Briefly, rats were anesthetized in a chamber using 3% isoflurane and a mixture of 70% nitrous oxide and 30% oxygen. Then rats were then transferred to a surgical table, where anesthesia was maintained with a facemask that delivered 1% isoflurane from a calibrated precision vaporizer, and poly-L-lysine-coated nylon (4.0) sutures were used to generate infarcts with minimal inter-animal variability. During the unilateral, 2-h MCAO procedure, cerebral blood flow (CBF), blood pCO_2_ and pO_2_, mean arterial pressure (MAP), and rectal temperature were monitored continuously. Rectal temperatures were maintained between 36.5°C and 37.5°C using a heating pad and a heating lamp. Ipsilesional ischemic cerebral hemispheres were used for molecular analysis.

### Treadmill Exercise

Animals were randomly assigned either to the exercise groups or the non-exercise control group. Exercise animals were run on a four-lane treadmill (ZS-PT-II, ZS Dichuang Instruments, Inc., Beijing, China), either at a constant speed of 30 m/min for 30 min each day (intense); or at 5 m/min for the first 10 min, 9 m/min for the second 10 min, and 12 m/min for the last 10 min on days 1 and 2, followed by 12 m/min on the third and subsequent days (mild). The mild exercise was begun at a shorter intensity (days 1–2) and ultimately ended with the final mild speed at 3 days and thereafter, such that low intensity was maintained throughout. This gradual start could not be achieved for the intense exercise group, however, as we would, in this case, have been unable to accurately assess the effects of high intensity in the short-term (3 days) when rats were sacrificed. Both exercise and non-exercise animals were housed in groups of three in standard cages for equal time.

### Neurological Deficit

The modified scoring systems proposed by Zea Longa (5-point) and Belayev et al. (1996) (12-point) were used to examine the severity of neurological deficits in rats before and after 24 h reperfusion (Li et al., 2019). After MCAO, rats with scores of 2 or below were considered to represent the unsuccessful establishment of the MCAO model and were consequently excluded (about 10%); exclusion was then confirmed on autopsy by lack of a core, indicating a faulty surgery.

### Cerebral Infarct Volume

At 3 days of ischemia and reperfusion in rats that underwent MCAO, brains were resected and cut into 2-mm-thick slices, which were then treated with 2,3,5-triphenyltetrazolium chloride (TTC; Sigma–Aldrich, St. Louis, MO, USA) for staining (Li et al., 2017b), facilitating the use of an indirect method for calculating infarct volume to minimize error caused by edema.

### Apoptotic Cell Death

For quantification of apoptosis-related DNA fragmentation, a commercial enzyme immunoassay was used to determine cytoplasmic histone-associated DNA fragments (Cell Death Detection ELISA; mlbio, Shanghai, China). The degree of apoptosis was quantified according to the amount of cytoplasmic histone-associated DNA fragments in the control and experimental groups at 3 days after physical exercise.

### Neurobehavioral Tests

These tests included adhesive removal, beam balance, forelimb placing, grid walking, and Rota-rod performance (R03–1; Xin-Ruan Instruments, Inc., Shanghai, China), as assessed at days 1, 3, 7, 14, 21, and 28. The Morris water maze (ZS-II; ZS Dichuang Instruments, Inc., Beijing, China) was also employed, in our case at 24–28 days; this is following a previous report that showed no obvious suppressive effect on swimming at 24 days after the ischemic event (Ran et al., 2018). In the adhesive removal test, the tape was attached to the palmar surface of the forepaw, and the time taken for the first attempt to touch and to remove the tape was recorded. In the beam balance test, rats were placed on a narrow wooden beam (122 × 2.5 × 42 cm), and performance was scored from 0 to 6 (0 = no attempt to stay on the beam; 1 = attempted to stay on the beam but no movement; 2 = attempted to cross the beam but failed; 3 = crossed the beam with contralateral hindlimb slips >50% of the time; 5 = crossed the beam with contralateral hindlimb slips <50% of the time; 6 = crossed the beam without slips; see Ran et al. (2018). In the forelimb placing test, rats were held gently with forelimbs close to the tabletop while the surface was lightly brushed using each side of their vibrissa. The ability of rats to place the preferred forelimb on the edge of the table in this context was recorded 10 times, and placing rates were calculated. In the grid walk test, rats were placed on a wire grid (100 × 25 × 50 cm) and allowed to walk from one end to the other; the total number of foot slips during this crossing was recorded. In the Rota-rod test, rats were placed on a rotating drum that accelerated from 4 to 40 rpm within 300 s; the time that the animals stayed on the rotating rod was then recorded. Finally, in the Morris water maze, rats were placed into a pool (diameter = 150 cm) at one of the four locations, and allowed to swim for 90 s to find a hidden platform (diameter = 10 cm); swim speed and the time taken to find the hidden platform were recorded using a camera positioned above the pool that transmitted data to an analysis system for calculation. Tests were conducted on non-consecutive days to mitigate possible confounding due to motor learning that might have occurred if these tests were performed in close succession.

### Neuron Isolation and Flow Cytometry Assay

As described previously by our group (Chen et al., 2018), an adult brain dissociation kit (Miltenyi Biotec, Bergisch Gladbach, Germany) was used for neuron isolation. Briefly, rats were sacrificed at 3, 14, and 28 days after exercise and ipsilesional brains were finely cut, ground, and filtered through a 70-μm cell strainer (Miltenyi Biotec, Bergisch Gladbach, Germany) to obtain a single-cell suspension. Cell pellets (5 × 10^6^ cells) were stained with primary antibodies against Tau or GAP-43 (1 μg/1 × 10^6^ cells, rabbit anti-Tau, and rabbit anti-GAP43, Abcam, MA, USA) in darkness for 30 min at room temperature. Cells were washed three times with PBS and incubated with Alexa Fluor^®^ 488 fluorescein-conjugated secondary antibodies (Sigma, St. Louis, MO, USA) for 30 min at room temperature, and then washed again and analyzed on a FACS Calibur flow cytometer (Accuri C6, BD, San Jose, CA, USA) with Cell Quest software (BD, San Jose, CA, USA).

### Protein Expression

At 3, 14, and 28 days after initiation of the exercise regimens, rats were sacrificed for Western blot analysis. Tissue samples from the ipsilesional ischemic cerebral hemispheres of all experimental groups were harvested, and total protein extraction was performed using cell lysis solutions (Thermo Fisher Scientific, Inc., Waltham, MA, USA). Protein concentration was then determined by the BCA method. Electrophoresis (10% SDS-PAGE gel) was performed with 30 μg of protein per lane. Gel transfer to a PVDF membrane was performed under 200 V for 1 h. Membranes were blocked with 5% skimmed milk, followed by incubation with primary antibodies (1:1,000 rabbit anti-BDNF, rabbit anti-NGF, rabbit anti-PSD-95, rabbit anti-SYN, rabbit anti-Tau, and rabbit anti-GAP43, Abcam, MA, USA; 1:500 rabbit anti-HIF-1α, Santa Cruz Biotechnology, Inc., Santa Cruz, CA, USA) overnight at 4°C. The next day, membranes were washed three times and further incubated with a goat anti-rabbit IgG-HRP secondary antibody (1:1,000, Santa Cruz) at room temperature for 1 h. After washing, the ECL method was used to detect signals. Western blot images for each antibody were analyzed using an image analysis program (ImageJ 1.42, National Institutes of Health, Bethesda, MD, USA) to quantify protein expression according to relative image density.

### Statistical Analysis

Statistical analyses were performed with SPSS Statistics for Windows, Version 17.0 (SPSS Inc., Chicago, IL, USA). Differences among groups were assessed using one-way ANOVA with a significance level of *p* < 0.05. *Post hoc* comparison among groups was performed using the least significant difference method.

## Results

### Experimental Design and Physiological Parameters

**Figure 1:**
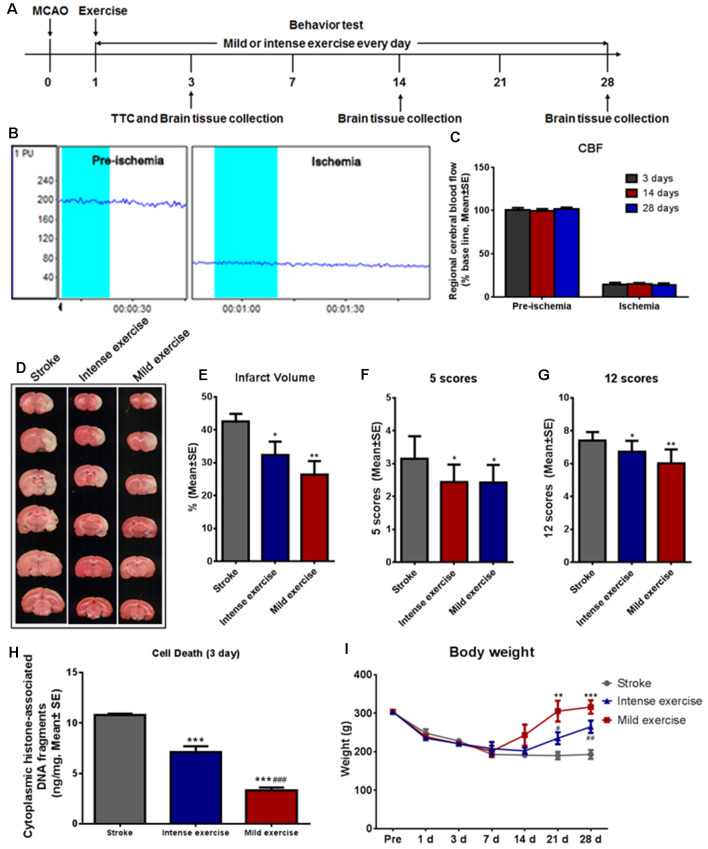
Mild or intense exercise reduced brain infarct. **(A)** Illustration of the experimental timelines. Rats were subjected to 2 h middle cerebral artery occlusion (MCAO), followed by daily treadmill exercise 1 day after reperfusion for up to 28 days. **(B,C)** Representative images and quantification of cerebral blood flow (CBF) monitoring of the three study groups for 2 min before and after the onset of ischemia. There were no significant differences in CBF between groups. **(D)** 2,3,5-triphenyltetrazolium chloride (TTC) histology demonstrating exercise-induced infarct volume reduction in the penumbra region of the ischemic territory supplied by the middle cerebral artery. **(E)** Quantification of the infarct volume reduction exercise. Both mild (**p < 0.01) and intense (*p < 0.05) exercise significantly decreased infarct volumes, but the reduction was more pronounced with mild exercise. Neurological deficits were tracked after both types of exercise using both the 5-** (F)** and 12-** (G)** point systems. ANOVA analyses indicated that both mild (**p < 0.01) and intense exercise (*p < 0.05) reduced neurological deficits. **(H)** Cell death reduction due to exercise quantified at 3 days. Both mild and intense exercise reduced apoptotic cell death significantly (***p < 0.001), but a more significant (^###^*p* < 0.001) decrease was shown in the mild exercise group. **(I)** Bodyweight was recorded at days 1, 3, 7, 14, 21 and 28. ** or *** Represent mild exercise vs. control; ^#^ or ^##^ represent intense exercise vs. control.

Illustration of the experimental timelines (Figure 1A). There were no significant differences in CBF (Figures 1B,C), blood MAP, pO_2_, or pCO_2_ (Table 1) between these groups.

**Table 1:** Physiological parameters during surgery.

	Stroke	Intense exercise	Mild exercise
MAP (mm Hg)			
Prior to MCAO	86.8 ± 3.3	87.0 ± 3.5	87.5 ± 3.4
Onset of reperfusion	86.7 ± 2.4	86.8 ± 2.5	86.4 ± 3.5
After reperfusion	82.3 ± 2.9	85.3 ± 3.1	85.8 ± 4.0
pCO_2_ (mm Hg)			
Prior to MCAO	44.8 ± 1.7	45.0 ± 2.2	47.2 ± 3.5
Onset of reperfusion	42.4 ± 2.7	45.4 ± 2.0	43.2 ± 2.1
After reperfusion	45.2 ± 4.0	44.3 ± 2.7	43.4 ± 4.4
pO_2_ (mm Hg)			
Before MCAO	132.9 ± 5.9	139.5 ± 5.6	132.4 ± 5.7
Onset of reperfusion	135.2 ± 5.1	132.7 ± 5.6	134.7 ± 4.9
After reperfusion	134.1 ± 9.1	138.2 ± 6.4	132.4 ± 4.1

*MAP, mean arterial pressure; MCAO, middle cerebral artery occlusion*.

### Brain Infarction and Correlates

A large infarct volume (42.5%) was seen following 2 h MCAO and 3 days reperfusion. Both mild (**p < 0.01) and intense (*p < 0.05) exercise significantly decreased infarct volumes (32.3% vs. 26.3%, respectively; Figures 1D,E). Neurological deficits were detected by the 5- (Figure 1F) or 12- (Figure 1G) point score systems; compared to the control group, deficits were decreased significantly (*p < 0.05) after either mild or intense exercise. Apoptotic cell death was detected at 3 days as described above; both mild and intense exercise significantly (***p < 0.001) decreased cell death (0.07 and 0.14 ng/ml, respectively, vs. 0.22 ng/ml), but a further significant decrease was noted (***p < 0.001) in the mild exercise group (Figure 1H). Also, a significant (***p < 0.001) increase in weight was seen in both exercise groups, with mild exercise rats demonstrating additional gain (Figure 1I).

### Functional Outcomes

**Figure 2:**
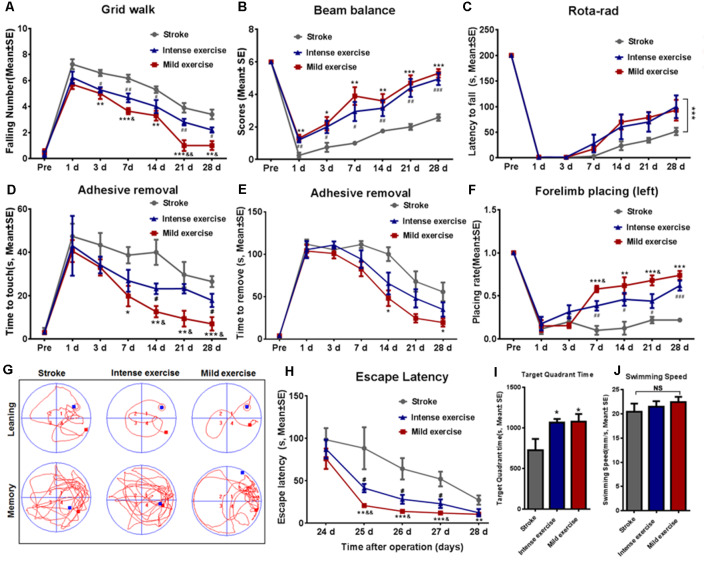
Exercise-mediated enhancement of functional recovery. **(A)** Grid walk test. Foot slips from the grid were significantly reduced after both mild (5.0 vs. 6.6 at 3 days, **p < 0.01; 3.6 vs. 6.2 at 7 days, ***p < 0.001; 3.3 vs. 5.3 at 14 days, **p < 0.01; 1.0 vs. 3.9 at 21 days, ***p < 0.001; 1.0 vs. 3.4 at 28 days, **p < 0.01) and intense (5.2 vs. 6.6 at 3 days, ^#^*p* < 0.05; 4.7 vs. 6.2 at 7 days, ^##^*p* < 0.01; 4.0 vs. 5.3 at 14 days, ^#^*p* < 0.05; 2.8 vs. 3.9 at 21 days, ^#^*p* < 0.05; 2.2 vs. 3.4 at 28 days, ^##^*p* < 0.01) exercise rats as compared to control rats at 3, 7, 14, 21, and 28 days. Mild exercise conferred further benefit over intense exercise in this respect (5.7 vs. 6.2 at 1 day; 5.0 vs 5.2 at 3 days; 3.6 vs. 4.7 at 7 days, ^&^*p* < 0.05; 3.3 vs. 4.0 at 14 days; 1.0 vs. 2.8 at 21 days, ^&&^*p* < 0.01; 1.0 vs. 2.2 at 28 days, ^&^*p* < 0.05). Similar results were observed in beam balance **(B)**, Rota-rod **(C)**, adhesive removal **(D,E)**, and forelimb placing tests** (F)**. Learning ability was examined by the Morris water maze test at 24–28 days of exercise **(G–J).** Representative images of the swim paths at 28 days **(G)**. Latency to locate the submerged platform at 24–28 days **(H)**. Target quadrant time **(I)** and swim speed **(J)** at 28 days. *p ≤ 0.05, **p ≤ 0.01, ***p ≤ 0.001 represent mild exercise vs. control; ^#^*p* ≤ 0.05, ^##^*p* ≤ 0.01, ^###^*p* ≤ 0.001 represent intense exercise vs. control; ^&^*p* ≤ 0.05 represent intense exercise vs. mild exercise. NS, not significant.

As shown in Figure 2A, the time taken to fall off the grid was significantly reduced after both mild (*p* < 0.01) or intense (*p* < 0.05) exercise as compared to rest at 3, 7, 14, 21, and 28 days (Figure 2A); this reduction was significantly more pronounced in mild exercise rats on 7, 21, and 28 days. Similar results were observed in beam balance (Figure 2B), Rota-rod (Figure 2C), adhesive removal (Figures 2D–E), and forelimb placing tests (Figure 2F). On assessment using the Morris water maze (Figures 2G–J) at 24–28 days, exercised rats demonstrated significantly shorter latency to locate the hidden platform as compared to rested controls, with mildly exercised rats attaining significantly better outcomes (Figures 2G–H). Exercised rats spent more time (*p < 0.05) in the target quadrant to find the hidden submerged platform than rested rats (Figure 2I). In contrast, there was no significant difference between groups concerning swim speed, suggesting similar gross motor skills (Figure 2J). These results demonstrate the significant role of exercise generally, and mild exercise in particular, in the long-term recovery of sensorimotor functions and spatial learning capability after ischemia/reperfusion injury.

### Neuroplasticity

**Figure 3:**
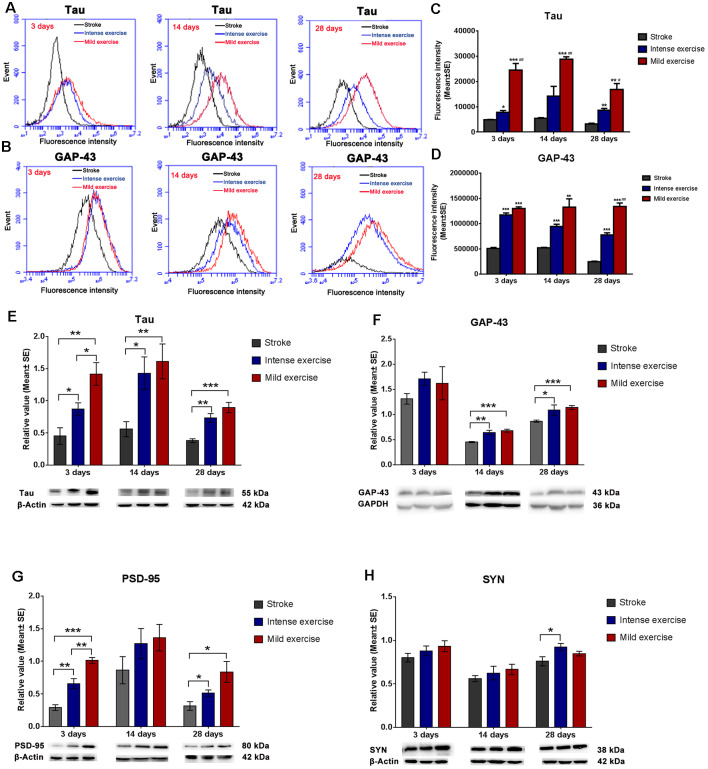
Exercise-induced increased expression of synaptic proteins.** (A–D)** Representative images of Tau and GAP-43 detected by FCM. Both mild (**A,C**; 24,600.2 vs. 4,885.12 at 3 days, ***p < 0.001; 28,897 vs. 5,408.9 at 14 days, ***p < 0.001; 16,879.2 vs. 3,186.86 at 28 days, **p < 0.01) and intense (7,752.19 vs. 4,885.12 at 3 days, *p < 0.05; 14,230.5 vs. 5,408.9 at 14 days; 8,698.12 vs. 3,186.86 at 28 days, **p < 0.01) exercise significantly induced Tau expression at 3, 14, and 28 days. Further increases in expression were seen in mildly exercised rats at 3, 14, and 28 days (^#^0.05, ^##^0.01, ^###^0.001 represent mild exercise vs. intense exercise). The same results were also seen for GAP-43 expression **(B,D)**. **(E–H)** Representative images of Tau, GAP-43, PSD-95, and SYN as detected by Western Blot. Compared to the control group, levels of Tau (**E**; 1.4 vs. 0.5 at 3 days, **p < 0.01; 1.6 vs. 0.6 at 14 days, **p < 0.01; 0.9 vs. 0.4 at 28 days, ***p < 0.001), GAP-43 (**F**; 1.6 vs. 1.3 at 3 days; 0.7 vs. 0.5 at 14 days, ***p < 0.001; 1.1 vs. 0.8 at 28 days, ***p < 0.001), PSD-95 (**G**; 1.0 vs. 0.3 at 3 days, ***p < 0.001; 1.4 vs. 0.9 at 14 days; 0.8 vs. 0.3 at 28 days, *p < 0.05), and SYN (**H**; 0.9 vs. 0.8 at 3 days; 0.7 vs. 0.6 at 14 days; 0.8 vs. 0.8 at 28 days) in mildly exercised rats were increased. The same results were also seen with intense exercise. Levels of Tau (**E**; 1.4 vs. 0.9 at 3 days, *p < 0.05; 1.6 vs. 1.4 at 14 days; 0.9 vs. 0.7 at 28 days), GAP-43 (**F**; 1.6 vs. 1.7 at 3 days; 0.7 vs. 0.6 at 14 days; 1.1 vs. 1.0 at 28 days), PSD-95 (**G**; 1.0 vs. 0.7 at 3 days, **p < 0.01; 1.4 vs. 1.3 at 14 days; 0.8 vs. 0.5 at 28 days), and SYN (**H**; 0.9 vs. 0.9 at 3 days; 0.7 vs. 0.6 at 14 days; 0.8 vs. 0.9 at 28 days) were similar between mildly and intensely exercised rats.

Flow cytometry assay demonstrated that both mild (**p < 0.01) and intense (**p < 0.01) exercise increased expression of Tau (Figures 3A,C) and GAP-43 (Figures 3B,D) at 3, 14, and 28 days; significantly more Tau expression was seen in mildly exercised rats (**p < 0.01). Also, compared to the control group, mild and intense exercise both significantly increased protein expression of Tau, GAP-43, and PSD-95 at 3, 14, and 28 days. Compared to the control group, levels of Tau (**p < 0.01, Figure 3E), GAP-43 (***p < 0.001, Figure 3F), PSD-95 (*p < 0.05, Figure 3G), and SYN (Figure 3H) were found by Western Blot to be increased in mildly exercised rats at 3, 14, and 28 days; the same results were also seen in the intense exercise group. Taken together, these results demonstrate the capacity of exercise to augment neuroplasticity after ischemia/reperfusion injury.

### Expression of HIF-1α, BDNF, TrkB, and CREB

**Figure 4:**
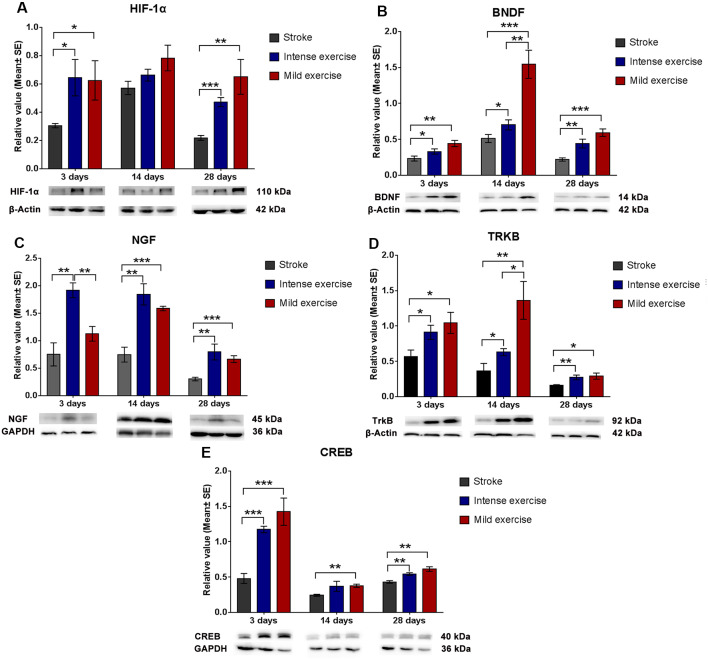
Augmented HIF-1α/BDNF/ TrkB/CREB pathway protein expression after exercise. Compared to rested rats, levels of HIF-1α **(A**; 0.6 vs. 0.3 at 3 days, *p < 0.05; 0.8 vs. 0.6 at 14 days; 0.7 vs. 0.2 at 28 days, **p < 0.01), BDNF **(B**; 0.4 vs. 0.2 at 3 days; 1.5 vs. 0.5 at 14 days; 0.6 vs. 0.2 at 28 days), NGF **(C**; 1.1 vs. 0.7 at 3 days; 1.6 vs. 0.7 at 14 days, ***p < 0.001; 0.6 vs. 0.3 at 28 days, ***p < 0.001), TrkB **(D**; 1.0 vs. 0.6 at 3 days, *p < 0.05; 1.4 vs. 0.4 at 14 days, **p < 0.01; 0.3 vs. 0.2 at 28 days, *p < 0.05), and CREB **(E**; 1.4 vs. 5.5 at 3 days, ***p < 0.001; 0.4 vs. 0.2 at 14 days, **p < 0.01; 0.6 vs. 0.4 at 28 days, **p < 0.01) in mildly exercised rats were significantly increased. The same results were seen in intensely exercised rats. Levels of HIF-1α **(A**; 0.6 vs. 0.6 at 3 days, *p < 0.05; 0.8 vs. 0.7 at 14 days; 0.7 vs. 0.5 at 28 days), BDNF **(B**; 0.4 vs. 0.3 at 3 days; 1.5 vs. 0.7 at 14 days, **p < 0.01; 0.6 vs. 0.4 at 28 days), NGF **(C**; 1.1 vs. 1.9 at 3 days, **p < 0.01; 1.6 vs. 1.8 at 14 days; 0.6 vs. 0.8 at 28 days), TrkB **(D**; 1.0 vs. 0.9 at 3 days; 1.4 vs. 0.6 at 14 days, *p < 0.05; 0.3 vs. 0.3 at 28 days), and CREB **(E**; 1.4 vs. 1.2 at 3 days; 04 vs. 0.4 at 14 days; 0.6 vs. 0.5 at 28 days) were similar between exercise intensities.

Both exercise protocols yielded a significant increase in levels of these proteins at 3, 14, and 28 days. Compared to the control group, levels of HIF-1α (3 days, *p < 0.05; 28 days, **p < 0.01, Figure 4A), BDNF (Figure 4B), NGF (14 days and 28 days, ***p < 0.001, Figure 4C), TrkB (*p < 0.05, Figure 4D), and CREB (**p < 0.01, Figure 4E) were significantly increased in both the mild and intense exercise groups. Levels of HIF-1α (3 days, *p < 0.05, Figure 4A), BDNF (14 days, **p < 0.01, Figure 4B), NGF (3 days, **p < 0.01, Figure 4C), TrkB (14 days, *p < 0.05, Figure 4D), and CREB (Figure 4E) were further increased in mildly exercised rats. These results demonstrate the alterations in HIF-1α, BDNF, TrkB, and CREB in association with synaptic plasticity following ischemia/reperfusion injury.

## Discussion

The results obtained in this study, confirmed the augmentation in neuroplasticity in the ipsilesional hemisphere and functional outcomes provided by physical exercise after ischemic brain injury. Specifically, we showed that both mild and intense exercise regimens reduced brain infarct volume and apoptotic cell death, and improved motor and cognitive function at 3, 14, and 28 days after ischemia/reperfusion injury. The early improvement in infarct volume seen in these results aligned with a previous meta-analysis, in which infarct volume was reduced most effectively by exercise administered with the shortest delays after ischemia (Egan et al., 2014); data from our group derived from pre-conditioning experimentation suggest that this may be related to the capacity of exercise to mitigate inflammatory damage during reperfusion (Ding et al., 2005), with the caveat that the exercise initiation too early after ischemia may be detrimental (Li et al., 2017a). More recent work by our group further substantiated these findings by demonstrating that exercise improved glycometabolism in the ischemic area and decreased neuroinflammation and apoptosis as early as 1 day post-stroke, and also at 3 days (Shen et al., 2016; Li et al., 2017a,b,c). These findings suggest that it is beneficial to initiate exercise early after ischemia/reperfusion, as was done in the present study.

Furthermore, our biochemical analyses showed that the expression of synaptic plasticity proteins (Tau, GAP-43, and PSD-95) and their potential upstream regulators (HIF-1α, BDNF, NGF, TrkB, and CREB) were significantly increased after exercise. These findings suggest that long-term physical exercise may induce synaptic plasticity through the HIF-1α and BDNF/TrkB/CREB pathway. Brain synaptic regeneration may be related to elevated levels of GAP-43 or Tau proteins, and exercise has been shown to increase expression of GAP-43 in the ischemic area in rats with cerebral ischemia/reperfusion injury (Mizutani et al., 2011). Exercise-induced GAP-43 has been associated with augmented hippocampal neuroplasticity (Liu et al., 2018; Rahmati and Kazemi, 2019) in a process that appears to be dependent on BDNF maturation and the TrkB signaling promoted by mature BDNF (Ding et al., 2011). Similarly, exercise has been shown to promote axonal recovery as assessed by the upregulation of Tau and GAP-43 and is associated with functional improvement after cerebral infarction (Li et al., 2015). Short-term moderate exercise also appears to be capable of inducing the BDNF-regulated marker of hippocampal and structural plasticity known as SYN (Ferreira et al., 2011). One critical component of synaptic plasticity, the dynamic reorganization of the PSD protein scaffold (Coley and Gao, 2019), is augmented by yet another synaptic protein, PSD-95. PSD-95 is constituent of the postsynaptic membrane that plays a key role in the plasticity and structure of the excitatory chemical synapse (Wu et al., 2017), and multiple studies have associated physical exercise with its induction (Jung and Kim, 2017; Pan et al., 2017). A series of studies have shed light on the relationship between these factors by demonstrating that BDNF/NGF participate in promoting neuroplasticity for motor rehabilitation after focal cerebral infarction (Matsuda et al., 2011; Mizutani et al., 2011; Mang et al., 2013); BDNF was reported to be induced by exercise, and may regulate the expression of synaptic proteins including GAP-43 (Liu W. et al., 2018), SYN (Ferreira et al., 2011), PSD-95 (Li X. et al., 2019) and Tau (Kerling et al., 2017). This research indicates that synaptic plasticity after stroke is determined, at least in part, by the induction and upregulation of axonal or synaptic proteins that, in our study, were found to be increased in both exercise cohorts.

Also, our work helps to elucidate the role occupied by another factor: HIF-1α. The present results are consistent with previous studies showed that the upregulation of HIF-1α promoted synapse plasticity by mediating synaptic markers (Li G. et al., 2019), and played a beneficial role in post-stroke exercise inducing angiogenesis and neurogenesis (Li C. et al., 2017). Recent work demonstrates that exercise could activate the cerebral motor and cognitive circuits by increasing the expression of HIF-1α, suggesting a regulatory role of HIF-1α in exercise-enhanced neuroplasticity (Halliday et al., 2019). Additionally, previous studies also indicated that HIF-1α regulated BDNF (Chen et al., 2017), TrkB (Martens et al., 2007), and CREB (Yu et al., 2020). Furthermore, the activation of the BDNF/TrkB/CREB pathway has been reported to contribute to the reduction in cerebral ischemic injury and improvement in functional recovery after stroke (Liu H. et al., 2018). Therefore, the enhanced expression of HIF-1α and BDNF/TrkB/CREB proteins after ischemia/reperfusion injury in the present study suggest that HIF-1α might be involved in the BDNF pathway, known to promote synaptic plasticity. Although, we did not explicitly study the regulation of HIF-1α on the BDNF/TrkB/CREB pathway, our results suggest a potential link between the molecules. Our findings could be a basis to further clarify the participation of HIF-1α in BDNF-mediated synaptogenesis.

The results obtained in this experiment are in agreement with previous studies showing that exercise improves motor and cognitive function (Chen et al., 2019; Palasz et al., 2019; Tíglás et al., 2019). Our results also, by suggesting the adequacy of milder post-stroke exercise, address the controversy in the literature regarding the dependence of these beneficial effects on the intensity of the prescribed exercise regimens (Han et al., 2017). A previous investigation supports the findings in the present study, by reporting that higher intensity exercise can exacerbate brain injury after ischemia, whereas the effects of mild intensity training were found to be encouraging (Scopel et al., 2006). Additional work has shown that cell proliferation in the dentate gyrus (Kim et al., 2003), spatial memory function (Lee et al., 2009), and synaptic plasticity (Shih et al., 2013) were more remarkable with mild rather than with heavy exercise after ischemia. In contrast, high-intensity intermittent exercise (HIT) was reported to be superior to moderate-intensity continuous training (MCT) in improving neural plasticity after cerebral ischemia in rats (Pin-Barre et al., 2017; Luo et al., 2019). HIT had a similar effect on cardiac troponin-I as workload-matched continuous exercise in endurance runners, which could be considered as high intensity exercise (Li et al., 2020) and was reported to be acceptable in stroke patients (Boyne et al., 2016). In agreement with these studies, our present results support the beneficial effect of intense exercise, but also indicate that mild exercise is not necessarily worse; and may be adequate to augment neuroprotection and neuroplasticity after stroke. Further investigation is needed in order to optimize exercise intensity post-stroke and determine which intensity may be most beneficial for neurorehabilitation. Furthermore, a standardized definition of exercise intensities may be necessary in order to homogenize methodologies and better compare results between studies in the future.

The protocol utilized in the present study to define exercise intensities was based on prior works (Curry et al., 2010; Zhang et al., 2012). We employed as a standard of achieved exercise training intensity the speed at which rats could not run any longer due to fatigue within three minutes after the onset of exercise. The therapeutic doses of physical exercise training used in our study were calculated as 40% of this maximum velocity in the case of mild exercise training, which amounted to approximately 15 m/min, and 80% in the case of intense exercise training, which was about 32 m/min (Zhang et al., 2012). To further increase the difference between our categories, we reduced speed in the mild group to a maximum of 12 m/min as previous studies (Tian et al., 2013; Zhang P. et al., 2013; Zhang et al., 2017; Tang et al., 2018). For the high-intensity group, we selected 30 m/min because we have employed this speed in previous work, in which we found that it reduced brain damage (Ding et al., 2006), blood-brain barrier dysfunction (Guo et al., 2008), and brain inflammation in stroke (Curry et al., 2010). Recently, studies used physiological parameters, transferable to patients, to determine high and low intensity in rats (Pin-Barre et al., 2017; Luo et al., 2019). To agree with the principle that a physical exercise regimen is reproducible (Gronwald et al., 2019), our further exercise procedure would focus on a physiological indicator such as the lactate threshold. Another key finding from our previous work was the influence of initiation time on post-stroke rehabilitation outcomes: initiation 6 h post-stroke exacerbated brain damage, but this was avoided when exercise was deferred for 1–3 days (Li et al., 2017a,b). Therefore, the initiation time of 24 h after stroke was selected in this study.

In this study, we intended to observe the protective effects of post-stroke exercise on brain infarct at 3 days as previous studies did. Recent work by our group substantiates these findings by demonstrating exercise-improved glycometabolism, decreased neuroinflammation, and apoptosis (Li et al., 2017a,b,c). The present results were largely supported by the findings of other groups, which demonstrated that exercise accelerated CBF (Pianta et al., 2019), decreased infarct volume (Tian et al., 2013; Zhang Y. et al., 2013; Pan et al., 2020) and improved functional outcomes (Pianta et al., 2019). In contrast, a few studies have reported no neuroprotective effects of exercise on the neurological deficit and infarct volume after stroke within 3 days (Matsuda et al., 2011; Cui et al., 2020). Future studies are necessary to fully elucidate the effect of post-stroke exercise on brain injury.

Some limitations are important to consider when interpreting the results of our study. The different constant-intensity regimens were used to demonstrate the concept of mild or intense exercise. Although the exercise protocol in rats cannot be directly transferred to patients, in the present study, as the first step, we intended to use these two exercise procedures to investigate the mechanism underlying the dose-dependent benefit of exercise on recovery after stroke. More careful design is on the way to develop a translational strategy that better applies to human stroke patients (Gronwald et al., 2019). For this purpose, our future work will focus on a connection between animal and clinical exercise procedures by controlling the workload of each training regimen as well as using lactate threshold or oxygen uptake as an indicator. Regarding the interpretation of the neurobehavioral test, possibly the multiple Rotarod tests served as a training procedure that may have influenced our results. Given our study’s focus on the effect and mechanism of different exercise doses on rehabilitation, in each group, the rats received the same test. It is unlikely that this small amount of possible training would change the direction of our results.

In conclusion, this study demonstrates the positive effect on brain injury, functional outcome, and neuroplasticity conferred by both mild and intense long-term treadmill exercise. Additionally, our results suggest that intense exercise did not confer further benefit when compared with its milder counterpart, thus mild exercise may be adequate and sufficient to elicit rehabilitative benefits post-stroke. Moreover, the results may provide a base for our future study regarding the regulation of HIF-1α on the BDNF/TrkB/CREB pathway in the biochemical processes underlying post-stroke synaptic plasticity.

## Data Availability Statement

All datasets generated for this study are included in the article/Supplementary Material.

## Ethics Statement

The animal study was reviewed and approved by the Animal Care and Use Committee of the Capital Medical University.

## Author Contributions

FL conducted the animal and biochemical experiments employed in this research. FL, XG, CH, CS, and YD were instrumental in preparing or revising the manuscript. YD was responsible for the experimental design, in addition to assisting with manuscript preparation and revision.

## Conflict of Interest

The authors declare that the research was conducted in the absence of any commercial or financial relationships that could be construed as a potential conflict of interest.
