# Supplementary material for: In Search of a Dose: The Functional and Molecular Effects of Exercise on Post-stroke Rehabilitation in Rats
Source: Front Cell Neurosci. 2020 Jun 25;14:186. doi: 10.3389/fncel.2020.00186 (PMC7330054; doi:10.3389/fncel.2020.00186)
Supplement: Supplementary file 1 [file Data_Sheet_1.PDF]

Figure s1

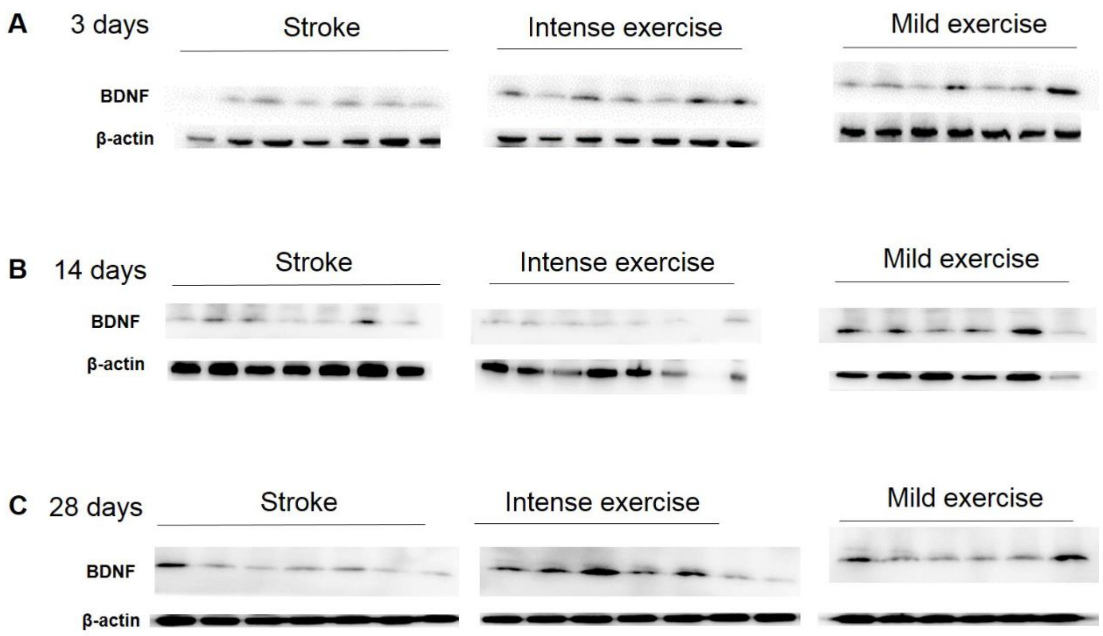

Figure s1. All samples used for the quantification of BDNF at 3days (A), 14 days (B), and 28 days (C).

Figure s2

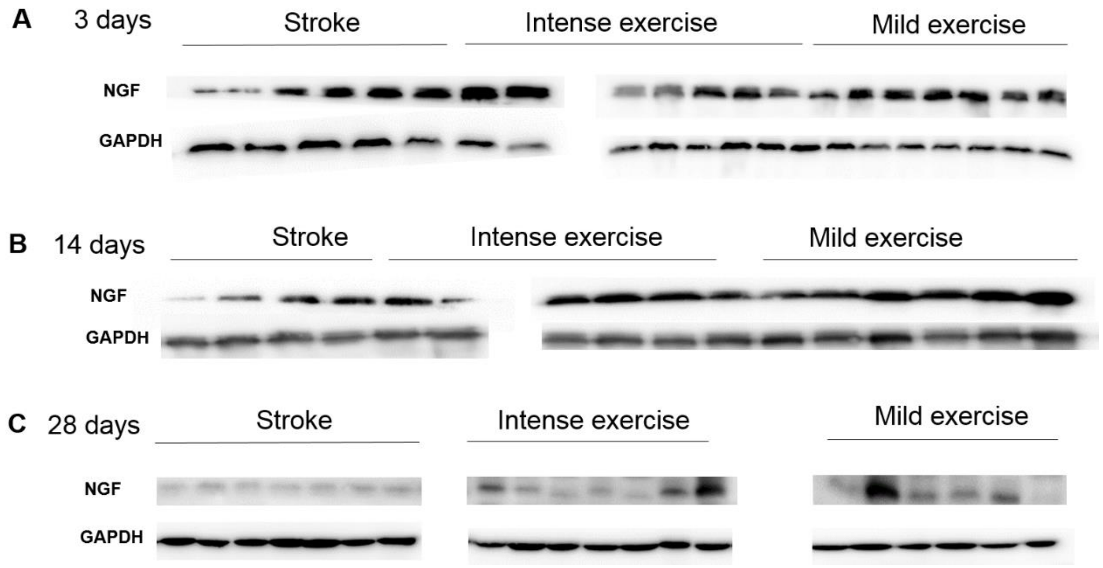

Figure s2. All samples used for the quantification of NGF at 3days (A), 14 days (B), and 28 days (C).

Figure s3

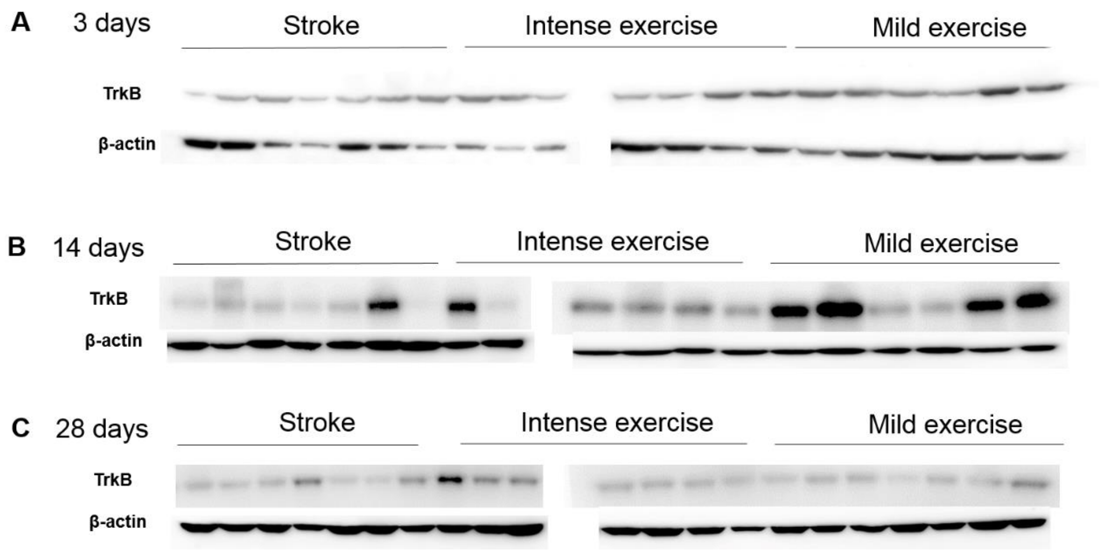

Figure s3. All samples used for the quantification of TrkB at 3days (A), 14 days (B), and 28 days (C).

Figure s4

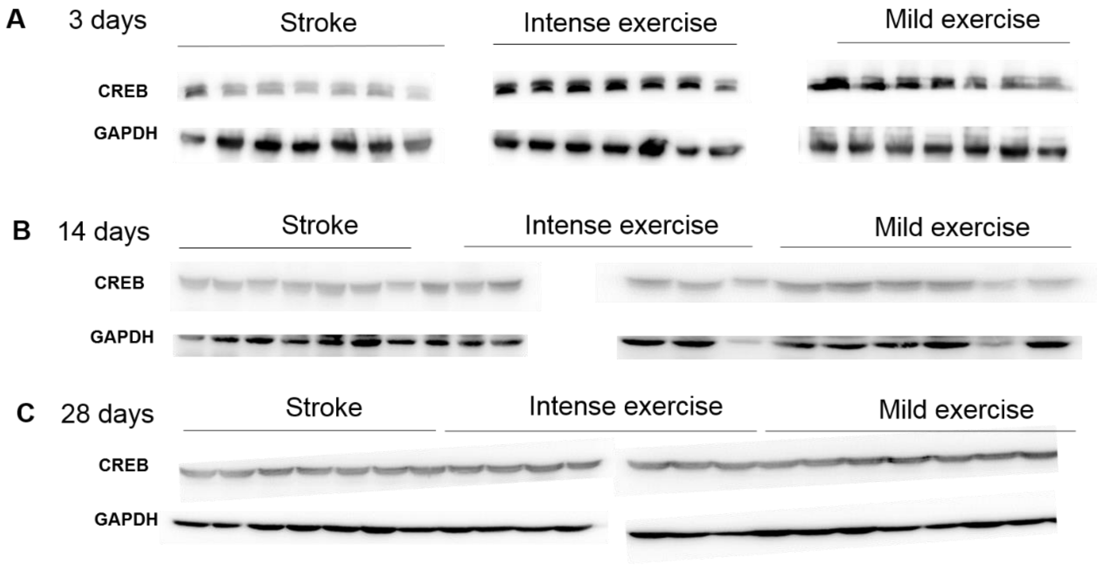

Figure s4. All samples used for the quantification of CREB at 3days (A), 14 days (B), and 28 days (C).

Figure s5

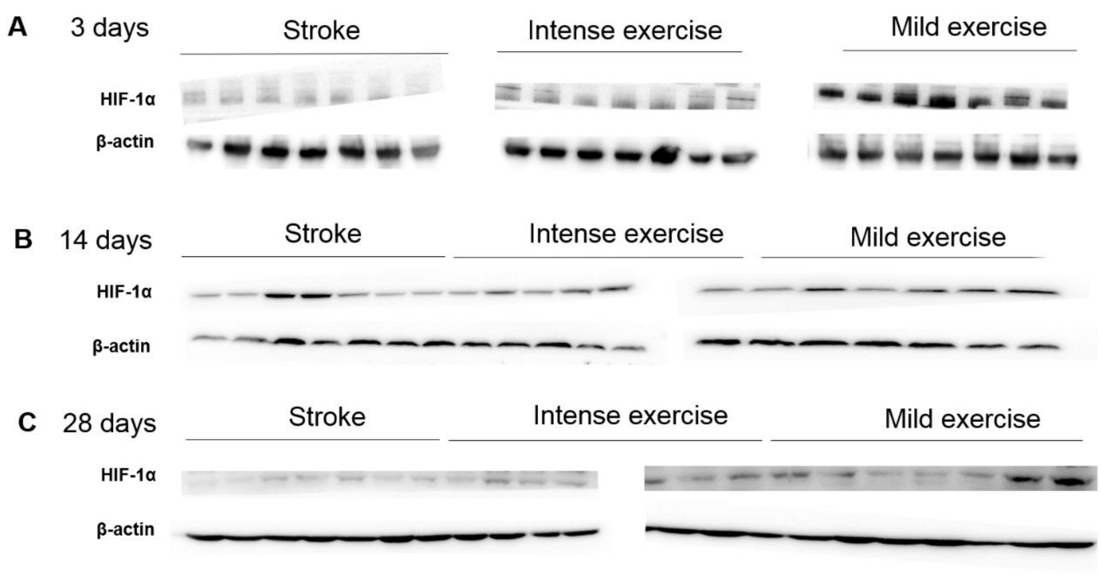

Figure s5. All samples used for the quantification of HIF1-α at 3days (A), 14 days (B), and 28 days (C).
